# Oral antioxidant therapy for juvenile rats with kaolin-induced hydrocephalus

**DOI:** 10.1186/2045-8118-11-23

**Published:** 2014-10-13

**Authors:** Domenico L Di Curzio, Emily Turner-Brannen, Marc R Del Bigio

**Affiliations:** 1 Departments of Human Anatomy & Cell Science, 715 McDermot Avenue, Winnipeg, MB R3E 3P4, Canada; 2 Manitoba Institute of Child Health, 715 McDermot Avenue, Winnipeg, MB R3E 3P4, Canada; 3 Department of Pathology, University of Manitoba, 727 McDermot Avenue, Winnipeg, R3E 3P5, Canada

**Keywords:** Rodent model, Brain, Hydrocephalus, Antioxidant, Oxidative stress

## Abstract

**Background:**

Oxidative and nitrosylative changes have been shown to occur in conjunction with the hypoxic changes and cellular/axonal damage in hydrocephalic rodent brains. We hypothesized that antioxidant therapy would improve behavioral, neurophysiological, and/or neurobiochemical outcomes in juvenile rats following induction of hydrocephalus.

**Methods:**

Three-week old rats received an injection of kaolin (aluminum silicate) into the cisterna magna. Magnetic resonance (MR) imaging was performed two weeks later to assess ventricle size and stratify rats to four treatment conditions. Rats were treated for two weeks daily with sham therapy of either oral canola oil or dextrose or experimental therapy of a low or high dose of an antioxidant mixture containing α-tocopherol, L-ascorbic acid, coenzyme Q10 (CoQ10), reduced glutathione, and reduced lipoic acid. Behavior was examined thrice weekly.

**Results:**

All hydrocephalic groups lagged in weight gain in comparison to non-hydrocephalic controls, all developed significant ventriculomegaly, and all exhibited white matter destruction. Canola oil with or without the antioxidant mixture normalized antioxidant capacity in brain tissue, and the dextrose-treated rats had the greatest ventricular enlargement during the treatment period. However, there were no significant differences between the four treatment groups of hydrocephalic rats for the various behavioral tasks. Glial fibrillary acidic protein and myelin basic protein quantitation showed no differences between the treatment groups or with control rats. There was increased lipid peroxidation in the hydrocephalic rats compared to controls but no differences between treatment groups.

**Conclusion:**

The antioxidant cocktail showed no therapeutic benefits for juvenile rats with kaolin-induced hydrocephalus although canola oil might have mild benefit.

## Background

Hydrocephalus is a neurological condition in which altered cerebrospinal fluid (CSF) flow dynamics lead to expansion of ventricular cavities in the brain. The neuropathophysiological damage associated with hydrocephalus is multifactorial with mechanical factors and reduced white matter blood flow simultaneously contributing to axonal and oligodendroglial damage [1,2]. Hypoxic changes in proteins of white matter glial and endothelial cells have been found in hydrocephalic rats using immunohistochemical detection of pimonidazole [3]. Oxidative stress leads to cell damage in various neurodegenerative disorders, including Parkinson disease, Alzheimer disease, Huntington disease, amyotrophic lateral sclerosis, and multiple sclerosis [4-8]. Lipid peroxidation, which follows oxygen free radical damage to cell membranes, has been shown in rodent hydrocephalic brains by measuring malondialdehyde (MDA) levels using the biochemical assay of thiobarbituric acid reaction substances (TBARS) [3,9-11] and a lipid peroxidation assay kit [12]. Immunohistochemical detection of MDA and 4-hydroxy-2-nonenal (4-HNE) [3] and superoxide dismutase (SOD) [13] and a fluorometric assay for reactive oxygen species (ROS), using dichlorofluorescein fluorescence [12], have also shown evidence of oxidation in hydrocephalic rat brain tissue.

Symptomatic hydrocephalus is typically treated by shunting, where CSF is diverted to another body site [14]. Although early CSF shunting shows some benefits in restoring periventricular white matter damage and cerebral blood flow, axonal damage is irreversible [1,2,15,16]. Moreover, treatment by shunting is associated with frequent complications, particularly obstruction and infection in young infants, which add to its morbidity and mortality [17,18]. With such potential complications, current methods of treatment need improvement, which warrants the need for novel therapeutic interventions to supplement shunt surgeries. In the juvenile rat model, parenteral administration of nimodipine or magnesium sulfate [19,20] and continuous intraventricular infusion of decorin [21] has some protective value. Experimental hydrocephalus was induced by kaolin (aluminum silicate) injections into the cisterna magna at 3 weeks in the rats in these and other studies in our laboratory [3,22-24] because the rat brain at this age is developmentally similar to that of a human infant [25-27].

Because oxidative stress is associated with brain damage in hydrocephalus, antioxidant therapy has been considered a potential pharmacological treatment for this condition. N-acetylcysteine [10] and the main constituent of green tea polyphenols, epigallocatechin gallate [11] have been shown to reduce lipid peroxidation when injected daily into the peritoneum of rats with experimentally-induced hydrocephalus at 7 days and 3 weeks, respectively. Antioxidants have shown beneficial outcomes in treating other neurological disorders. In particular, oral coenzyme Q10 (CoQ10) dietary supplementation in humans and animals is found to reduce oxidative stress by decreasing lipid peroxidation and may be a neuroprotectant in Parkinson disease, atherosclerosis, ischemia, toxic brain injury, and Alzheimer disease [28,29]. Alpha/gamma tocopherol (vitamin E) added to drinking water has been shown to reduce brain infarct volume after middle cerebral artery occlusion focal ischemia in rats [30] and is functionally interrelated with other antioxidants, including CoQ10, ascorbic acid, glutathione, and lipoic acid [31]. Oral treatment with the combination of α-lipoic acid and vitamin E can decrease lipid peroxidation and astroglial and microglial reactivity in the cerebrum of ischemic rats [32]. Oral CoQ10 and ascorbic acid (vitamin C) have been used to enhance oxidative phosphorylation in patients with mitochondrial disorders [33]. These antioxidant agents alone and in combination have low toxicity and have been shown to be of potential value in animal and human studies when given orally. Thus, we hypothesized that oral treatment with a combination of these antioxidant agents would lead to behavioral and/or structural improvements in rats induced with experimental hydrocephalus using kaolin injections.

## Methods

### Animals

Locally bred Sprague–Dawley (Trial 1, n = 45) and Long Evans rats (Trial 2, n = 52) were housed in conventional cages with four animals per cage. Pelleted food and water were provided ad libitum. The housing room was kept on a 12:12 h (6 a.m. – 6 p.m.) light–dark cycle, and the room temperature was 21–23**°**C. For identification, the rats were ear punched. All animals were treated humanely in accordance with guidelines set forth by the Canadian Council on Animal Care. The University of Manitoba Bannatyne Campus (Animal) Protocol Management & Review Committee approved the experimental protocols.

### Hydrocephalus induction

At 21–23 days of age (weight 42-88 g) rats were anesthetized using 3% isoflurane in oxygen. The back of the neck was shaved, the skin was cleaned with chlorhexidine followed by 70% alcohol, and then a sterile 27-gauge needle (shallow bevel “blunt” tip) was inserted percutaneously into the cisterna magna. A sterile suspension of kaolin (aluminum silicate, 250 mg/mL; Sigma, St. Louis MO) in 0.9% normal saline (0.04 mL) was injected slowly to induce hydrocephalus (n = 89). Littermate controls (n = 8) received sham injections of sterile saline solution. Following recovery from the anesthetic, the rats were observed for signs of neurological impairment. Subcutaneous buprenorphine (0.03 mg/kg) was administered every 12 hours for 2 days to control pain. Rats were weighed daily. Those experiencing severe impairment or weight loss >15% of the starting weight were euthanized.

### Magnetic resonance imaging and assignment to treatment groups

*T2*–weighted magnetic resonance imaging (MRI) was performed as previously described [20,22] on day 13 or 14 post injection. The ventricle to brain area ratio was performed using computerized planimetry with Marevisi (NRC, Winnipeg, MB, Canada) and Image J software on a coronal image of the cerebrum just caudal to the optic chiasm. The size of frontal horn of the lateral ventricles was calculated by dividing the area of the ventricles by the area of the cerebrum. Rats were stratified according to ventricle size and assigned listwise to vehicle sham, dextrose sham, low dose, or high dose dietary intervention treatment groups. A second set of MR images was obtained 2 weeks later, no more than 24 hours prior to euthanasia. For each rat, the ventricle to brain area ratio was measured again and compared to the images obtained prior to treatment to determine the relative change in ventricle size within treatment groups.

### Drug preparation and administration

The antioxidant therapy was performed separately with two trials of rats (n = 45 and n = 52, respectively). The treatment procedures were the same for both trials except where indicated. All animals were weighed daily, and their weights were used to determine treatment volumes. The rats received these calculated dosages daily for two weeks by oral gavage to ensure that all animals consumed the entire dosage. Daily gavage with 1 ml saline was started 1 week prior to kaolin injection so that the rats were habituated to the intervention. The rats in the first trial received their oral treatment in the late afternoon after all behavioral testing was completed, whereas the rats in the second trial received their treatment in the early morning before any behavioral testing was performed.

A combination of antioxidant agents shown to be effective in a variety of neurological disease models was chosen. Alpha-tocopherol (Sigma T3251) at 50 and 250 mg/kg, L-ascorbic acid (Sigma A7506) at 20 and 100 mg/kg [34,35], CoQ10 (Sigma C9538) at 40 and 200 mg/kg [31], reduced glutathione (Sigma G4251) at 20 and 100 mg/kg [36], and reduced lipoic acid (Sigma T8260) at 20 and 100 mg/kg [37] provided the antioxidant ingredients for the low and high dose concentrations, respectively. These were made up in a mixture of 0.45% saline with 30% canola oil. Ingredients were blended thoroughly at room temperature and stored in dark containers at 4°C. In the first trial, the control rats received saline solution with 5% dextrose added to make up for the calories in the treatment groups. In addition, because of supply problems, the first trial rats received the reduced lipoic acid only during the first week. For the second trial, the low and high doses were made up separately and stored in different containers. The control hydrocephalic rats received a 0.20 mL/100 g volume of either saline solution with 5% dextrose or the vehicle of 30% canola oil (which was also given to nonhydrocephalic controls).

### Behavioral testing

All behavioral testing was completed in a blinded manner. The rats performed various tests including mobility, stance, and gait in the open field, gait on a rotarod at steady and accelerating speed, swim speed in a narrow pool, memory in a modified water maze test as assessed previously [19,20,22], and gait on a modified ladder test [38]. They were always tested in the same order, where open field and rotarod tests were done on one day, the ladder test on the next day, and the water-related tasks on the third day weekly. Testing began the same week of kaolin and sham injections and was performed for 4 weeks, i.e., during the 2 weeks after injections and the 2 weeks of antioxidant or sham therapy. During the first trial, all quantitative behavioral measurements were obtained by manual timing using a stopwatch only. For the second trial, water maze behavior was videotaped and analyzed using HVS Image 2100 Plus Tracking System software (HVS Image Ltd, Twickenham, Middlesex, UK). The ladder test was only performed during the second trial.

### Histopathological and biochemical studies following drug treatments

Rats were euthanized within 24 hours of final MR imaging using isoflurane anesthesia (5%) followed by carbon dioxide gas narcosis and exsanguination. Blood was flushed by transcardiac perfusion with ice-cold 0.1 M phosphate-buffered saline (PBS), and the brains quickly removed. Cerebral hemispheres were split in the midline. The left side was divided into dorsal frontal cerebrum, dorsal parietal cerebrum, hippocampus, and cerebellum then frozen in liquid nitrogen and stored at -70°C. The right side was immersion fixed in cold 3% buffered paraformaldehyde and after 3–5 days was cut coronally, dehydrated, and embedded in paraffin wax.

Paraffin sections (6 μm thickness) of the cerebrum at the level of the anterior horn of the lateral ventricles were stained with hematoxylin & eosin Y (H&E) and solochrome cyanine & eosin for visualization of myelin. Sections were immunostained with rabbit polyclonal anti-glial fibrillary acidic protein (GFAP) (1:12800 dilution; DAKO Z0334; Glostrup, Denmark) to label astrocytes. The primary antibody underwent 1–2 hour incubation at room temperature. This was followed by incubation with appropriate biotinylated secondary antibody, followed by reaction with streptavidin-peroxidase, detection with diaminobenzidine (DAB, Sigma D5905), and finally counterstaining with hematoxylin. Negative controls were treated without the primary antibody. Corpus callosum thickness was measured using 400x ocular magnification at the sagittal midline and above the lateral angle of the frontal horn of the lateral ventricle medial to the external capsule. The latter site was chosen because the midline region was often disrupted preventing proper measure and comparison to nonhydrocephalic animals.

Frozen frontal and parietal cerebrum samples were homogenized using a RIPA buffer with protease inhibitors phenylmethylsulfonyl fluoride (PMSF) and aprotinin. Total protein quantification was performed using the Micro BCA™ (Pierce) Protein Assay kit (Thermo Scientific, Rockford, Illinois, USA). Homogenates were used to quantitate myelin basic protein (MBP) and GFAP by using enzyme-linked immunosorbent assays (ELISA), as previously described in detail [39,40]. Assays were performed in triplicate. Results for the first trial are given in mg GFAP per gram of protein and averaged for all dilutions, while the second trial averaged results per sample.

### TBARS and total antioxidant assays

Lipid peroxidation was measured to determine if hydrocephalic rat brains had increased oxidative byproduct formation. Dorsal frontal and parietal cerebrum homogenates (second trial only) were tested using the thiobarbituric acid reactive substances (TBARS) assay at pH 3.5; TBA reacts with malondialdehyde (MDA), which is a secondary product of lipid peroxidation, yielding a product that can be detected spectrophotometrically at 532 nm [3,41]. The assay was performed as previously described with a few modifications, and samples were run in triplicate. The standard, 1′1′3′3 tetramethoxypropane (TMP) (Sigma Cat #108383), was dissolved initially with dimethyl sulfoxide (DMSO) to 1 M concentration and then diluted in distilled water to 2.5-50 μM. In addition, 1-butanol and pyridine were not added after heating the TBA solution at 95**°**C for one hour, as the TBA and MDA red pigmented reaction product was evident during the heating process.

The Antioxidant Assay Kit (Sigma Cat #CS0790) was used to measure total antioxidant levels in brain tissue (second trial only). The frozen hippocampus was mechanically homogenized in ice-cold 1x Assay Buffer as instructed (Sigma Cat # A3605). The assay is based on the reaction between hydrogen peroxide (H_2_O_2_) and metmyoglobin; the ferryl myoglobin radical oxidizes 2,2′-azino-bis (3-ethylbenzthiazoline-6-sulfonic acid) (ABTS) to form a radical cation (ABTS^**+**^), which is detected by spectrophotometry at 405 nm. Samples were run in duplicate.

### Statistical analysis

Unless otherwise stated, all data are presented as mean ± standard error of the mean (SEM). Quantitative data were analyzed to confirm a normal distribution. For all analyses, *p* values ≤ 0.05 were deemed statistically significant. Statistical analyses were conducted separately for the first (n = 45) and second (n = 52) trials of rats, which consisted of ANOVA with post-hoc analyses conducted for some measures using the Bonferroni-Dunn multiple inter-group comparisons approaches where indicated. Non-parametric score data were analyzed with Mann–Whitney *U* test or Kruskal-Wallis test for two or three groups, respectively. For the second trial, qualitative assessments for the sham control and hydrocephalic rats were analyzed separately from quantitative measures. Two-tailed Student’s *t*-tests were conducted for behavioral testing, ventricle size, histological data, biochemical, and ELISA values to compare the control and hydrocephalus groups. Statistical analyses were conducted using the SPSS 14.0 software program.

## Results

### Mortality

No animals died during kaolin injection. Of the 89 rats that were given a kaolin-injection at three weeks age, 4 were euthanized approximately two weeks post-injection before the onset of therapy and 1 died 2 days before the end of treatment because of severe neurological deficits. The remaining rats underwent the two-week antioxidant therapy regime according to the stratification of treatment conditions and were sacrificed at seven weeks of age. Age-matched control rats (n = 8) were also euthanized at seven weeks age and 24 hours post-MRI.

### Ventricle size on magnetic resonance imaging

**Figure 1:**
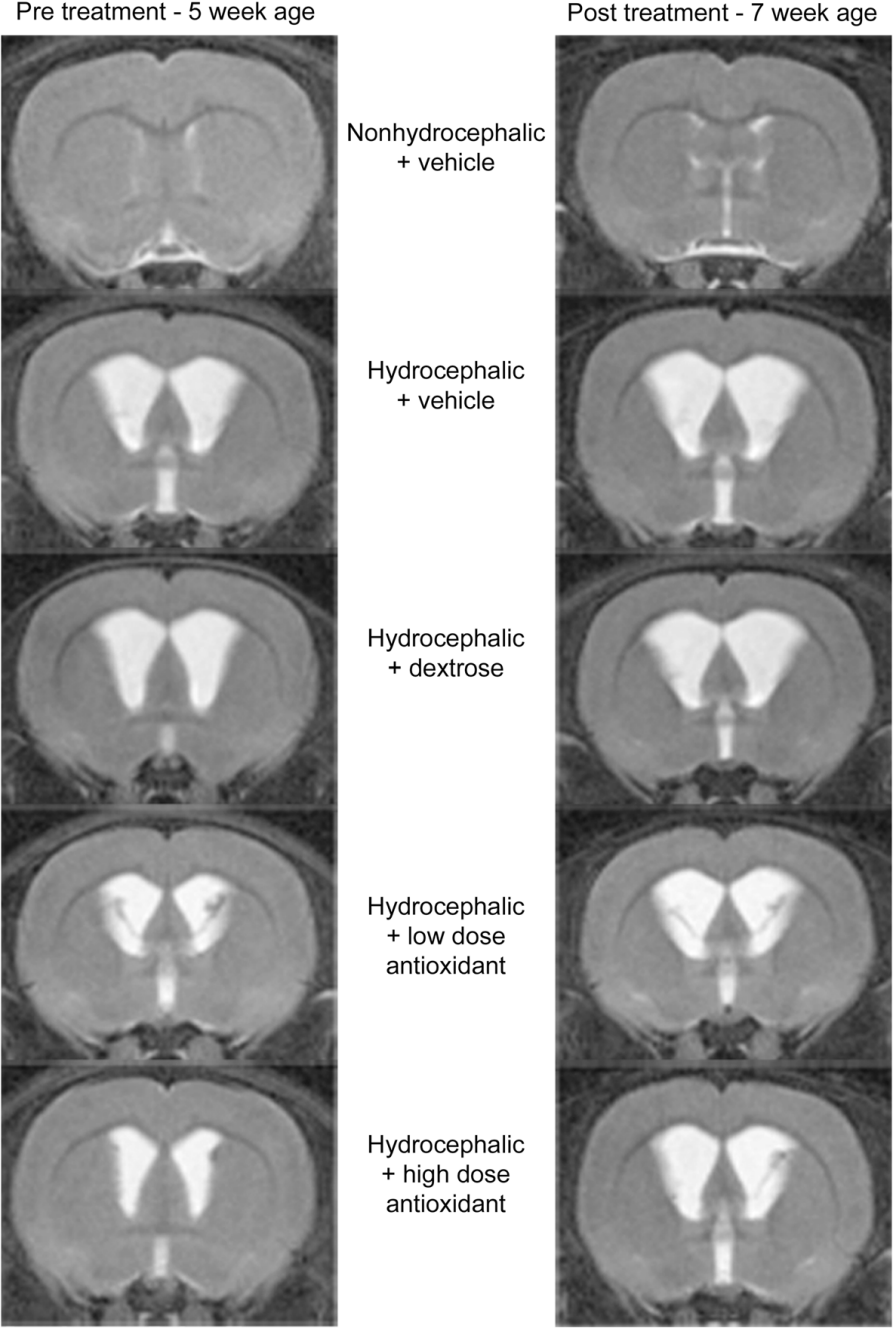
**Magnetic resonance (MR) images displaying progressive hydrocephalus in rats at 5 and 7 weeks of age that underwent injection of kaolin into the cisterna magna at 3 weeks.** These *T2*-weighted images depict the coronal view of the cerebral cortex at the level of the frontal horn of the lateral ventricle where cerebrospinal fluid (CSF) is white (bright) in the lateral and third ventricles and subarachnoid space (SAS). The control rat images are shown at the top, and the ventricles are very narrow. Ventricular enlargement is obvious in all hydrocephalic rats before treatment, and all treated groups displayed further dilatation after treatment.

The first MR images showed that kaolin injections into the cisterna magna lead to dilatation of the cerebral ventricles in five-week old rats (Figure 1). In both trials, there was no significant difference between the groups prior to onset of antioxidant therapy. All groups showed continued enlargement of the ventricles during the therapeutic period, and all groups displayed significant increases in lateral ventricle size compared to the images before treatment began (all *p* < 0.05, *t*-tests; Tables 1 and 2; Figure 2). In the first trial, the high dose treatment groups had the most severe ventricular enlargement with a 33.5% increase after treatment (Table 1). In the second trial, the low and high dose groups showed less ventricular enlargement than dextrose control (*p* = 0.012 and 0.041, respectively), but there was no benefit above canola oil vehicle-treated hydrocephalic rats (Table 2).

**Table 1:** Results of antioxidant treatment on hydrocephalic rats (Trial 1)*

	**Dextrose**	**Low dose antiox.†**	**High dose antiox.†**
Sample size	14	14	15
Ventricle area index (pre-treat)	0.130 ± 0.010	0.128 ± 0.008	0.145 ± 0.021
Ventricle area index (post-treat)	0.141 ± 0.012#	0.138 ± 0.009#	0.198 ± 0.039#
Percent enlargement ventricles during treatment	9.3 ± 3.1	8.6 ± 2.0	33.5 ± 18.7
Body weight (g) (pre-treat)	106.1 ± 2.4	103.7 ± 1.1	106.6 ± 3.3
Body weight (g) (post-treat)	223.8 ± 4.5	223.1 ± 3.2	222.8 ± 7.0
Rearing activity (post-treat) (beam breaks in 5 min)	108 ± 18	146 ± 18	166 ± 26
Ambulatory activity (post-treat) (beam breaks in 5 min)	760 ± 85	824 ± 87	1284 ± 221
Total activity (post-treat) (beam breaks in 5 min)	1089 ± 107	1210 ± 107	1684 ± 248
Endurance of rotarod (cont. speed, post-treat) (sec)	60 ± 12	64 ± 11	44 ± 11
Endurance of rotarod (accelerating speed, post-treat) (sec)	21 ± 6	27 ± 6	25 ± 8
Morris water maze test (post-treat, 3 trials mean) (sec)	15.98 ± 1.21	15.17 ± 1.82	33.86 ± 12.17
Swim time 150 cm (post-treat) (sec)	11.98 ± 1.31	16.45 ± 1.87	14.31 ± 1.78
GFAP content (ELISA) frontal cerebrum (mg GFAP/g protein)	1.30 ± 0.13	1.54 ± 0.31	1.56 ± 0.32
MBP content (ELISA) frontal cerebrum (mg MBP/g protein)	5.19 ± 0.19	5.31 ± 0.12	5.24 ± 0.25

All data are presented as mean ± SEM.

*There were no significant differences between the treatment groups for any of the results in Trial 1.

†The low and high dose antioxidant combinations were made up in 0.45% saline with 30% canola oil.

#*p <* 0.05 before (pre-treat) vs. after (post-treat) treatment, Student’s *t*-tests.

**Table 2:** Results of antioxidant treatment on hydrocephalic rats (Trial 2)

	**Nonhydrocephalic controls**	**Vehicle (30% canola)**	**Dextrose**	**Low dose antiox.†**	**High dose antiox.†**
Sample size	8	11	10	11	10
Ventricle area index (pre-treat)	0.006 ± 0.0007	0.114 ± 0.013**	0.119 ± 0.016**	0.107 ± 0.015**	0.118 ± 0.015**
Ventricle area index (post-treat)	0.012 ± 0.003	0.140 ± 0.018**#	0.169 ± 0.027**#	0.125 ± 0.013**#	0.140 ± 0.010**#
Percent enlargement ventricles during treatment	-	22.5 ± 8.1	40.1 ± 6.2	23.0 ± 6.8	25.1 ± 8.3
Body weight (g) (pre-treat)	169.3 ± 4.6	123.3 ± 7.2**	135.2 ± 8.2**	116.9 ± 6.4**	112.7 ± 5.3**
Body weight (g) (post-treat)	269.3 ± 6.7#	200.2 ± 13.4**#	219.4 ± 13.5*#	175.4 ± 12.5**#	179.6 ± 12.0**#
Rearing activity (post-treat) (beam breaks - 5 min)	100 ± 12	67 ± 12	96 ± 8	78 ± 16	82 ± 16
Ambulatory activity (post-treat) (beam breaks - 5 min)	466 ± 35	334 ± 31*#	548 ± 54#	461 ± 39#	396 ± 53#
Total activity (post-treat) (beam breaks - 5 min)	618 ± 41	459 ± 38*#	713 ± 54#	637 ± 41#	558 ± 53#
Endurance of rotarod (cont. speed, post-treat) (sec)	33 ± 8	32 ± 10	37 ± 7	66 ± 15#	38 ± 11
Endurance of rotarod (accelerating speed, post-treat) (sec)	57 ± 15	56 ± 11#	73 ± 14#	65 ± 13#	71 ± 13#
Morris water maze test (post-treat, 3 trials mean) (sec)	7.67 ± 0.85#	6.98 ± 1.10#	5.89 ± 0.61#	5.15 ± 0.41*#	4.46 ± 0.46**#
Swim time 150 cm (post-treat) (sec)	24.3 ± 6.7	13.4 ± 2.0	20.9 ± 3.4	18.0 ± 3.4#	17.0 ± 3.1
Lateral corpus callosum thickness (μm)	315 ± 16	208 ± 14**	220 ± 20**	220 ± 18**	208 ± 13**
GFAP content (ELISA) frontal cerebrum (mg GFAP/g protein)	0.68 ± 0.16	1.08 ± 0.19	1.29 ± 0.24	1.11 ± 0.16	1.10 ± 0.25
MBP content (ELISA) frontal cerebrum (mg MBP/g protein)	2.30 ± 0.26	2.42 ± 0.28	2.91 ± 0.28	2.70 ± 0.35	2.30 ± 0.16
MDA level (TBARS) parietal cerebrum (μM MDA/g wet tissue)	111.98 ± 10.57	145.32 ± 8.94*	138.99 ± 9.41	146.89 ± 9.67*	141.35 ± 8.45*
ABTS + content (antioxidant capacity) hippocampus (mM)	0.580 ± 0.029	0.582 ± 0.025	0.467 ± 0.062	0.589 ± 0.019	0.574 ± 0.026

All data are presented as mean ± SEM.

†The low and high dose antioxidant combinations were made up in 0.45% saline with 30% canola oil.

*p < 0.05 control rats vs. hydrocephalic groups, Student’s *t*-tests or ANOVA with Bonferroni-Dunn post hoc tests for intergroup comparisons.

**p < 0.005 control rats vs. hydrocephalic groups, Student’s *t*-tests or ANOVA with Bonferroni-Dunn post hoc tests for intergroup comparisons.

#*p <* 0.05 before (pre-treat) vs. after (post-treat) treatment, Student’s *t*-tests.

**Figure 2:**
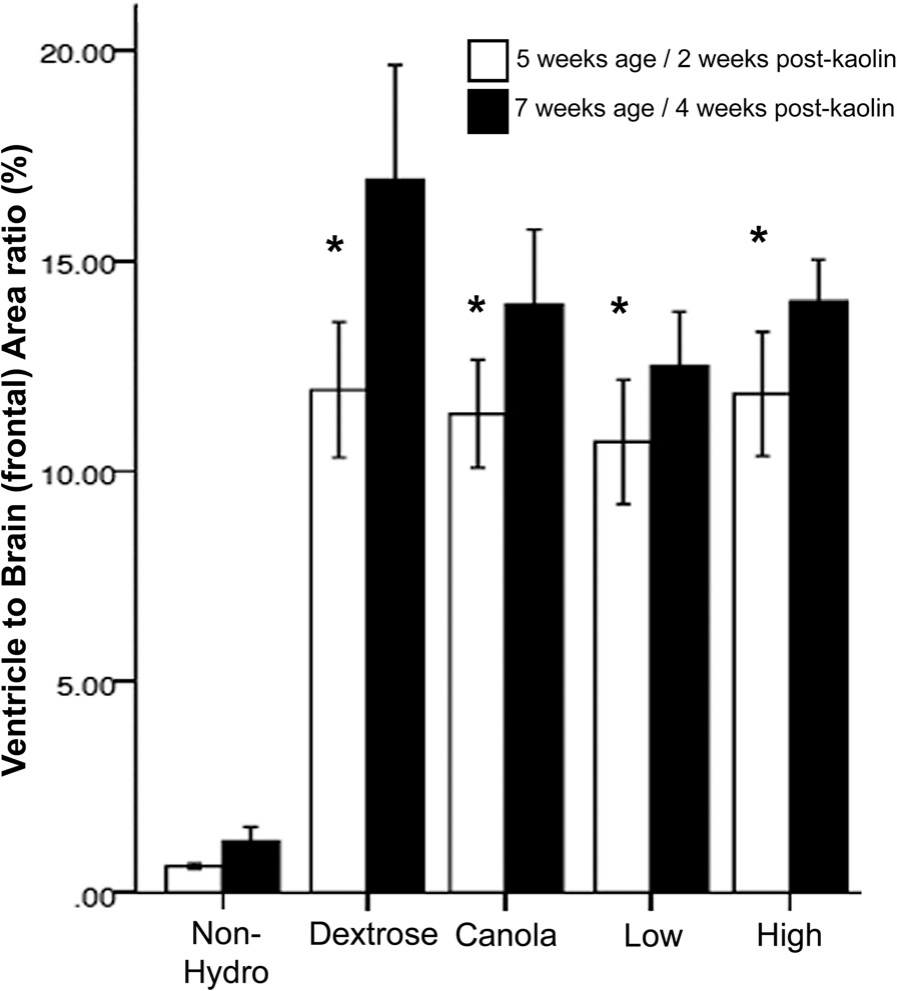
**Bar graph showing the lateral ventricles to frontal cerebrum brain area ratios (%) from the MR images for the nonhydrocephalic control rats compared to the four hydrocephalic treatment groups before and after antioxidant therapy.** All hydrocephalic groups had significantly enlarged ventricles compared to the nonhydrocephalic rats before and after treatment. * - Indicates significant increases in lateral ventricles to brain area ratios after treatment compared to before at *p* < 0.05.

### Behavioral assessments

Hydrocephalic rats gained less weight than nonhydrocephalic rats. They had hunched posture and unsteady gait on open field observations (as previously reported), although the quantitative measures of motor behavior only inconsistently showed deficits, the most obvious being reduced rearing activity (Table 2). In trial 1, there were no differences in the behavioral outcomes of hydrocephalic rats following 2 weeks of low or high dose antioxidant therapy compared to dextrose controls (Table 1). Post-hoc analyses showed that the high dose antioxidant treatment group displayed higher ambulatory movement compared to sham-treated rats after therapy that was approaching significance (*p* = 0.0575, Bonferroni test).

In the second trial, the four hydrocephalic treatment groups all weighed significantly less than nonhydrocephalic rats from 24 days of age to when they were sacrificed at 49 days of age (all *p* < 0.01, *t*-tests; ANOVA; Table 2). Despite the fact that the dextrose fed hydrocephalic controls had more severe ventricle enlargement, they tended to show greater weight gain. Only rearing behavior was significantly reduced as a consequence of hydrocephalus before treatment compared to nonhydrocephalic controls (*p* = 0.004, ANOVA). There was no consistent evidence for normalization of behavior abnormalities (ambulation, water maze, ladder test) associated with hydrocephalus following any of the treatments (Table 2).

### Structural and biochemical changes in brain

Histological examination corresponded to the MR imaging. There was appreciable thinning and fragmentation of periventricular white matter regions in all hydrocephalic animals. Corpus callosum thickness was significantly reduced overall in the hydrocephalic rats compared to control animals (*p* = 0.005, ANOVA). The lateral corpus callosum had more pronounced (32%) decrease in thickness (Table 2) than the medial corpus callosum (22%), and it was significantly reduced in all treatment groups compared to nonhydrocephalic controls (all *p* < 0.005, *t*-tests). Cortical and subcortical neuronal populations were not obviously damaged in the hydrocephalic rats compared to controls, unless the adjacent white matter was completely obliterated. Antioxidant therapy had no effect on corpus callosum thickness. Although the corpus callosum was thinned, overall the cerebral MBP content (relative to total protein) was not reduced as a consequence of hydrocephalus suggesting that surviving axons remained myelinated. The antioxidant treatment had no effect on MBP content (Tables 1 and 2).

In the frontal cortex at the level of the anterior horns of the lateral ventricles, sham and antioxidant-treated hydrocephalic rats displayed GFAP immunostaining of astrocytes in the perivascular and periventricular regions. There were no appreciable differences in GFAP 2expression between the hydrocephalic groups. In comparison to nonhydrocephalic controls, all had hypertrophic reactive astrocytes in the periventricular white matter and deep cortical regions. GFAP content, measured by ELISA, was increased in comparison to nonhydrocephalic controls, but there was no evidence that antioxidant therapy reduced the astroglial reaction (Tables 1 and 2). In the hindbrain surrounding the site of injection, there were no obvious inflammatory or reactive changes visible, such as macrophages, except in the most severe hydrocephalic rats that had to be sacrificed before the end of the antioxidant treatment (data not shown).

Lipid peroxidation in the parietal cerebrum, measured with the TBARS assay, was higher in hydrocephalic rats than in nonhydrocephalic controls. Comparing the four hydrocephalic treatment groups separately, all except the dextrose-treated group (*p* = 0.074) displayed significantly higher levels of oxidative stress (all *p* < 0.05) than nonhydrocephalic controls (Table 2). However, they were not significantly different from each other (*p* = 0.926, ANOVA; *t*-tests) suggesting that the oral antioxidant therapy had no beneficial effect.

Total antioxidant capacity of the hippocampal tissue, detected indirectly through the ABTS^+^ radical cation, displayed no appreciable differences between nonhydrocephalic controls compared to hydrocephalic rats. There were no significant differences in antioxidant levels between the four hydrocephalic treatment groups (*p* = 0.079, ANOVA); the dextrose-treated group exhibited the lowest values, but it was not significantly lower than the vehicle-treated group (*p* = 0.090, *t*-test) or nonhydrocephalic controls (*p* = 0.150, *t*-test) (Table 2).

## Discussion

Hydrocephalus was induced in three-week old rats, and oral antioxidant therapy was introduced two weeks later for a period of two weeks. Because oxidative stress occurs in hydrocephalus [3,9-13], it was hoped that antioxidants might have therapeutic benefits for treating hydrocephalus. The main positive outcome (in trial 2) was normalization of antioxidant capacity in brain tissue when hydrocephalic rats were administered canola oil with or without the antioxidant combination in comparison to the hydrocephalic rats that received dextrose. Canola oil components are known to have potent antioxidative capacity [42]. The dextrose treated rats had the greatest ventricular enlargement. Overall, however, there was no other behavioral, histological, or biochemical evidence to support the hypothesis that oral antioxidant therapy protects the hydrocephalic brain.

Progressive ventricular expansion has been a consistent finding in the pharmacological studies conducted in our lab using the kaolin induction model in rats [19,20,43-45]. We hoped the antioxidant therapy would display beneficial outcomes in stabilizing ventricular expansion or reducing associated brain damage and reactive changes like the results from Botfield *et al*. [21] using decorin treatment in juvenile hydrocephalic rats, but consistent findings were not observed in the two trials. In the first trial, the high dose antioxidant group exhibited the largest expansion of lateral ventricles overall, but this was due primarily to 1–2 outliers with severe ventriculomegaly. The second trial suggests that canola oil alone might have shown a benefit in reducing or stabilizing ventricular enlargement if the treatment period had been longer and the ventricles were allowed to reach a larger size. However, corpus callosum thickness was significantly reduced in the hydrocephalic rats, suggesting that further ventricular expansion would likely lead to increased destruction of this important white matter structure. Regardless, corpus callosum reduction was not ameliorated by antioxidant therapy, as opposed to prior drug trials from this lab showing that magnesium sulfate and nimodipine treatment of hydrocephalic rats reduces corpus callosum damage [19,20]. Minocycline-treated hydrocephalic rats are reported to have significantly increased cerebral cortex thickness in some brain regions compared to untreated rats [46]. Antioxidant therapy was also not associated with reduced GFAP immunoreactivity or content when compared to sham-treated hydrocephalic rats. However, magnesium sulfate, decorin, and minocycline have shown capacity to reduce reactive astrogliosis in hydrocephalic rats [20,21,46]. In addition, decorin has been shown to decrease activated microglia, macrophages, and other inflammatory cells accumulation, along with ventriculomegaly in hydrocephalic rats [21]. Astrocytic and microglial reactivity have also been reduced in rats with cerebral ischemia using the antioxidants α-lipoic acid and vitamin E [32], so it is uncertain why these agents, which possess synergistic effects, were unsuccessful at decreasing the reactive astrogliosis in our hydrocephalic rats. Memantine, a non-competitive NMDA receptor antagonist, administered for 2 weeks by intraperitoneal injection was shown to partially protect hippocampal neurons in 3-week old rats with kaolin-induced hydrocephalus, although no behavioral or ventricle size data were reported [47].

As expected, the current study showed increased oxidative stress in the hydrocephalic rats using the TBARS assay of lipid peroxidation [41,48]. Although the TBARS assay has been known to display other TBA reactive substances, previous studies using experimentally-induced or inherited hydrocephalus in rats have also shown increased oxidative stress when measuring lipid peroxidation, oxygen free radicals, and/or nitric oxide (NO) production [3,9-13]. Two studies showed that antioxidant treatment with intraperitoneal N-acetylcysteine [10] or epigallocatechin gallate [11] were potentially neuroprotective by reducing lipid peroxidation levels in cerebral and periventricular white matter regions, respectively. While the findings of these studies are promising, it is important to note that their scope is limited because lipid peroxidation was the only outcome parameter measured. In addition, no means were employed to confirm induction of hydrocephalus before antioxidant treatment began. This all must be taken into consideration when moving to human clinical trials as these drugs have shown only marginal success in rodent models of disease. It is of particular importance when considering the recommendations for success in treating neurological diseases, such as stroke or ischemic change in a clinical setting [49,50].

One potential shortcoming of our experiment was drug combination chosen. As described in the Introduction, all of the chosen agents have been effective orally in some experimental settings. Oral therapy offers longer periods of exposure, but peak levels are lower than those that follow parenteral administration. Furthermore, the extent to which the agents crossed the blood–brain barrier (BBB) is not clear. This is important because the BBB is generally intact in hydrocephalus [51]. Although all agents used in our antioxidant combination have been suggested to be orally efficacious in treating mitochondrial and neurodegenerative disorders, there are conflicting data concerning their ability to enter brain tissue. Glutathione is known to cross the BBB [36,52]. Zhang et al. [53] indicated that α-tocopherol is not restricted from the brain, while Gonzalez-Perez et al. [32] suggested that vitamin E and lipoic acid treatment for cerebral ischemia in rats may have only produced partial neuroprotective effects because of insufficient levels of these antioxidants in brain tissue. Oral administration of CoQ10 has been shown to elevate its levels in rat cerebral cortex mitochondria in one study [54], whereas others reported restricted uptake [31,53,55]. CoQ10 treatment via intraperitoneal injection also failed to show neuroprotective effects when administered to rats with induced focal and global ischemia [56]. More convincing evidence of the pharmacokinetics of CoQ10 was found by Sikorska et al. [57], who showed a complex of CoQ10 and an α-tocopherol derivative is neuroprotective in cerebral ischemia.

As noted above, 30% canola oil vehicle alone seemed to offer some benefit over dextrose therapy. Canola oil concentration was the same for both low and high dose antioxidant combinations. This potentially therapeutic effect could be related to the fact that canola oil augments entry of dietary vitamins C and E into brain tissue [58], or due to the potent scavenger 4-vinyl-2,6-dimethoxyphenol (canolol), which is the most active component in canola oil [42]. It scavenges both alkylperoxyl radicals (ROO ·) and peroxynitrite [59]. Peroxynitrite is a potent oxidizing and nitrating agent, and overproduction of it may be related to inflammation and neurodegenerative diseases [60,61]. Nitrite concentration is significantly increased in hydrocephalic brains [3], and nitric oxide can nitrate aromatic amino acids possibly through generation of peroxynitrite [62]. Thus, canolol in canola oil plays both antinitrosylating and antioxidative roles, which is more potent than α-tocopherol and vitamin C [42] that were used in our antioxidant combination. If confirmed in gyrencephalic species with larger brains, canola therapy would be simpler to use and less expensive than the drug combination tested here.

It should also be mentioned that in these experiments, the rats did not exhibit extreme ventriculomegaly overall. Consequently, the behavioral deficits were small and in some cases, there were no differences between control and hydrocephalic animals whether or not they received any antioxidant treatment. The behavioral results showed differences between trials for some of the tasks; this is likely related to the different rat strain and slightly different methods used to obtain the data. Despite this, with the rotating cylinder and the water maze tasks, there were no differences among the treatment conditions for both trials, which all showed improved performance after treatment. Reduced rearing activity was the only decrement observed in the hydrocephalic rats, with the exception of the dextrose-treated group, which paradoxically had the most severe ventriculomegaly. Perhaps only more severe damage (e.g. longer duration or greater ventriculomegaly) is associated with measurable behavioral deficits that might be amenable to pharmacologic intervention. The MBP content measured by ELISA showed discrepancy between trials. Possible explanations include the different rat strains used, slight differences in the age at time of sacrifice, and slight differences in the dissection of the dorsal brain sample, which was done by separate individuals in the two trials. Regardless, neither trial showed significant differences between the sham and treatment groups for MBP content.

## Conclusions

In summary, we did not find any behavioral, morphological, or biochemical evidence that oral antioxidant combination therapy provided appreciable therapeutic benefit to juvenile rats with kaolin-induced hydrocephalus. Potential explanations are the limited entry of the drugs into brain or the possibility that oxidative changes in hydrocephalic brains represent an end point marker of damage rather than the cause of the axonal damage. It should be noted that the canola oil vehicle alone did seem to normalize brain tissue antioxidant capacity and reduce the rate of ventricle enlargement in comparison to dextrose treatment. This safe and simple intervention should be confirmed.

## Abbreviations

4-HNE: 4-hydroxy-2-nonenal; ABTS: 2,2′-azino-bis (3-ethylbenzthiazoline-6-sulfonic acid); BBB: Blood–brain barrier; CoQ10: Coenzyme Q10; CSF: Cerebrospinal fluid; DAB: Diaminobenzidine; ELISA: Enzyme linked immunosorbent assay; GFAP: Glial fibrillary acidic protein; H&E: Hematoxylin and eosin; MBP: Myelin basic protein; MDA: Malondialdehyde; MR: Magnetic resonance; NRC: National Research Council; PBS: Phosphate-buffered saline; ROS: Reactive oxygen species; SEM: Standard error of the mean; SOD: Superoxide dismutase; TBARS: Thiobarbituric acid reaction substances; TE: echo time; TMP: 1′1′3′3 tetramethoxypropane; TR: recovery time.

## Competing interests

The authors declare that they have no competing interests.

## Authors’ contributions

DDC carried out the drug administration, behavioral work, dissections and histology, tissue homogenization and ELISAs, TBARS and Total Antioxidant assays, statistical analyses, and drafted the manuscript. DDC also participated in the induction of hydrocephalus and MR imaging. ETB participated in the tissue homogenization and ELISAs, TBARS and Total Antioxidant assays, and data collection. MDB conceived of the study including its design and coordination, carried out the hydrocephalus induction, and helped to draft the manuscript. All authors read and approved the final manuscript.

## References

[B1] Del Bigio M Neuropathology and structural changes in hydrocephalus Develop Disabil Res Rev 2010 16 16 22 10.1002/ddrr.94 20419767

[B2] McAllister J II Pathophysiology of congenital and neonatal hydrocephalus Sem Fet Neonat Med 2012 17 285 294 10.1016/j.siny.2012.06.004 22800608

[B3] Del Bigio M Khan OH da Silva Lopes L Packiasamy ARJ Cerebral white matter oxidation and nitrosylation in young rodents with kaolin-induced hydrocephalus J Neuropathol Exp Neurol 2012 71 4 274 288 10.1097/NEN.0b013e31824c1b44 22437339

[B4] Butterfield D Oxidative stress in neurodegenerative disorders Antioxid Redox Signal 2006 8 11–12 1971 1973 17034342 10.1089/ars.2006.8.1971

[B5] Emerit J Edeas M Bricaire F Neurodegenerative diseases and oxidative stress Biomed Pharmacother 2004 58 1 39 46 10.1016/j.biopha.2003.11.004 14739060

[B6] Simonian N Coyle JT Oxidative stress in neurodegenerative diseases Annu Rev Pharmacol Toxicol 1996 36 83 106 10.1146/annurev.pa.36.040196.000503 8725383

[B7] Sun A Chen YM Oxidative stress and neurodegenerative disorders J Biomed Sci 1998 5 401 414 10.1007/BF02255928 9845843

[B8] Uttara B Singh AV Zamboni P Mahajan RT Oxidative stress and neurodegenerative diseases: a review of upstream and downstream antioxidant therapeutic options Curr Neuropharm 2009 7 65 74 10.2174/157015909787602823 PMC2724665 19721819

[B9] Caner H Atasever A Kilinç K Durgun B Peker S Ozcan OE Lipid peroxide level increase in experimental hydrocephalus Acta Neurochir (Wien) 1993 121 1–2 68 71 8475810 10.1007/BF01405185

[B10] Etus V Gazioglu N Belce A N-acetylcystein reduces cerebral lipid peroxidation in a rat model of infantile hydrocephalus J Neurol Sci (Turkish) 2001 18 1 #2

[B11] Etus V Altug T Belce A Ceylan S Green tea polyphenol (-)-epigallocatechin gallate prevents oxidative damage on periventricular white matter of infantile rats with hydrocephalus Tohoku J Exp Med 2003 200 4 203 209 10.1620/tjem.200.203 14580151

[B12] Socci D Bjugstad KB Jones HC Pattisapu JV Arendash GW Evidence that oxidative stress is associated with the pathophysiology of inherited hydrocephalus in the H-Tx rat model Exp Neurol 1999 155 1 109 117 10.1006/exnr.1998.6969 9918710

[B13] Mori K Miyake H Kurisaka M Sakamoto T Immunohistochemical localization of superoxide dismutase in congenital hydrocephalic rat brain Childs Nerv Syst 1993 9 136 141 8374918 10.1007/BF00272261

[B14] Pudenz R The surgical treatment of hydrocephalus: an historical review Surg Neurol 1981 15 1 15 26 10.1016/S0090-3019(81)80084-5 7256520

[B15] Del Bigio M Cellular damage and prevention in childhood hydrocephalus Brain Pathol 2004 14 3 317 324 15446588 10.1111/j.1750-3639.2004.tb00071.x PMC8095857

[B16] Eskandari R McAllister JP II Miller JM Ding Y Ham SD Shearer DM Way JS Effects of hydrocephalus and ventriculoperitoneal shunt therapy on afferent and efferent connections in the feline sensorimotor cortex J Neurosurg 2004 101 2 Suppl 196 210 15835108 10.3171/ped.2004.101.2.0196

[B17] Epstein F How to keep shunts functioning, or “the impossible dream” Clin Neurosurg 1985 32 608 631 3905155

[B18] Riva D Milani N Giorgi C Pantaleoni C Zorzi C Devoti M Intelligence outcome in children with shunted hydrocephalus of different etiology Childs Nerv Syst 1994 10 1 70 73 10.1007/BF00313588 8194066

[B19] Del Bigio M Massicotte EM Protective effect of nimodipine on behavior and white matter of rats with hydrocephalus J Neurosurg 2001 94 5 788 794 10.3171/jns.2001.94.5.0788 11354411

[B20] Khan O Enno T Del Bigio MR Magnesium sulfate therapy is of mild benefit to young rats with kaolin-induced hydrocephalus Pediatr Res 2003 53 6 970 976 10.1203/01.PDR.0000061561.42921.5B 12621098

[B21] Botfield H Gonzalez AM Abdullah O Skjolding AD Berry M McAllister JP II Logan A Decorin prevents the development of juvenile communication hydrocephalus Brain 2013 136 2842 2858 10.1093/brain/awt203 23983032 PMC13375275

[B22] Del Bigio M Crook CR Buist R Magnetic resonance imaging and behavioral analysis of immature rats with kaolin-induced hydrocephalus: pre- and postshunting observations Exp Neurol 1997 148 256 264 10.1006/exnr.1997.6644 9398467

[B23] Del Bigio M Kanfer JN Zhang YW Myelination delay in the cerebral white matter of immature rats with kaolin-induced hydrocephalus is reversible J Neuropathol Exp Neurol 1997 56 9 1053 1066 10.1097/00005072-199709000-00010 9291946

[B24] Del Bigio M Zhang YW Cell death, axonal damage, and cell birth in the immature rat brain following induction of hydrocephalus Exp Neurol 1998 154 157 169 10.1006/exnr.1998.6922 9875277

[B25] Romijn H Hofman MA Gramsbergen A At what age is the developing cerebral cortex of the rat comparable to that of the full-term newborn human baby? Early Human Dev 1991 26 61 67 10.1016/0378-3782(91)90044-4 1914989

[B26] Tuor U Del Bigio MR Chumas PD Brain damage due to cerebral hypoxia/ischemia in the neonate: pathology and pharmacological modification Cerebrovasc Brain Metab Rev 1996 8 159 193 8727185

[B27] Clancy B Darlington RB Finlay BL Translating developmental time across mammalian species Neuroscience 2001 105 1 7 17 10.1016/S0306-4522(01)00171-3 11483296

[B28] Littarru G Tiano L Bioenergetic and antioxidant properties of coenzyme Q10: recent developments Mol Biotechnol 2007 37 1 31 37 10.1007/s12033-007-0052-y 17914161

[B29] Young A Johnson S Steffens DC Doraiswamy PM Coenzyme Q10: a review of its promise as a neuroprotectant CNS Spectr 2007 12 1 62 68 17192765 10.1017/s1092852900020538

[B30] Zhang B Tanaka J Yang L Yang L Sakanaka M Hata R Maeda N Mitsuda N Protective effect of vitamin E against focal brain ischemia and neuronal death through induction of target genes of hypoxia-inducible factor-1 Neuroscience 2004 126 433 440 10.1016/j.neuroscience.2004.03.057 15207361

[B31] Ibrahim W Bhagavan HN Chopra RK Chow CK Dietary coenzyme Q10 and vitamin E alter the status of these compounds in rat tissues and mitochondria J Nutr 2000 130 2343 2348 10958833 10.1093/jn/130.9.2343

[B32] Gonzalez-Perez O Gonzalez-Castaneda RE Huerta M Luquin S Gomez-Pinedo U Sanchez-Almaraz E Navarro-Ruiz A Garcia-Estrada J Beneficial effects of α-lipoic acid plus vitamin E on neurological deficit, reactive gliosis and neuronal remodeling in the penumbra of the ischemic rat brain Neurosci Let 2002 321 100 104 10.1016/S0304-3940(02)00056-3 11872266

[B33] Marriage B Clandinin MT Glerum DM Nutritional cofactor treatment in mitochondrial disorders J Am Diet Assoc 2003 103 1029 1038 10.1016/S0002-8223(03)00476-0 12891154

[B34] Faria R Abilio VC Grassl C Chinen CC Negrao LT de Castro JP Fukushiro DF Rodrigues MS Gomes PH Registro S de Carvalho Rde C D’Almeida V Silva RH Ribeiro Rde A Frussa-Filho R Beneficial effects of vitamin C and vitamin E on reserpine-induced oral dyskinesia in rats: Critical role of striatal catalase activity Neuropharmacology 2005 48 7 993 1001 10.1016/j.neuropharm.2005.01.014 15857626

[B35] Vatassery G Lai JC DeMaster EG Smith WE Quach HT Oxidation of vitamin E and vitamin C and inhibition of brain mitochondrial oxidative phosphorylation by peroxynitrite J Neurosci Res 2004 75 6 845 853 10.1002/jnr.20027 14994345

[B36] Sharma P Ahmad Shah Z Kumar A Islam F Mishra KP Role of combined administration of Tiron and glutathione against aluminum-induced oxidative stress in rat brain J Trace Elem Med Biol 2007 21 1 63 70 10.1016/j.jtemb.2006.12.001 17317527

[B37] Garcia-Estrada J Gonzalez-Perez O Gonzalez-Castaneda RE Martinez-Contreras A Luquin S de la Mora PG Navarro-Ruiz A An alpha-lipoic acid-vitamin E mixture reduces post-embolism lipid peroxidation, cerebral infarction, and neurological deficit in rats Neurosci Res 2003 47 2 219 224 10.1016/S0168-0102(03)00200-1 14512146

[B38] Metz G Whishaw IQ The ladder rung walking task: a scoring system and its practical application J Vis Exp 2009 28 e1204 10.3791/1204 PMC2796662 19525918

[B39] Di Curzio D Buist RJ Del Bigio MR Reduced subventricular zone proliferation and white matter damage in juvenile ferrets with kaolin-induced hydrocephalus Exp Neurol 2013 248 112 128 23769908 10.1016/j.expneurol.2013.06.004

[B40] Khan O Enno TL Del Bigio MR Brain damage in neonatal rats following kaolin induction of hydrocephalus Exp Neurol 2006 200 2 311 320 10.1016/j.expneurol.2006.02.113 16624304

[B41] Ohkawa H Ohishi N Yagi K Assay for lipid peroxides in animal tissues by thiobarbituric acid reaction Anal Biochem 1979 95 2 351 358 10.1016/0003-2697(79)90738-3 36810

[B42] Wakamatsu D Morimura S Sawa T Kida K Nakal C Maeda H Isolation, identification, and structure of a potent alkyl-peroxyl radical scavenger in crude canola oil, canolol Biosci Biotechnol Biochem 2005 69 8 1568 1574 10.1271/bbb.69.1568 16116287

[B43] Del Bigio M Wang X Wilson MJ Sodium channel-blocking agents are not of benefit to rats with kaolin-induced hydrocephalus Neurosurgery 2002 51 460 466 12182785 10.1097/00006123-200208000-00029

[B44] Khan O Enno T Del Bigio MR Tacrolimus and cyclosporine A are of no benefit to young rats with kaolin-induced hydrocephalus Pediatr Neurosurg 2003 39 309 313 10.1159/000075259 14734865

[B45] Khan O McPhee LC Moddemann LN Del Bigio MR Calcium antagonism in neonatal rats with kaolin-induced hydrocephalus J Child Neurol 2007 22 10 1161 1166 10.1177/0883073807306259 17940241

[B46] McAllister J II Miller JM Minocycline inhibits glial proliferation in the H-Tx rat model of congenital hydrocephalus Cerebrospinal Fluid Res 2010 7 1 7 10.1186/1743-8454-7-7 20507614 PMC2889858

[B47] Cabuk B Etus V Bozkurt SU Sav A Ceylan S Neuroprotective effect of memantine on hippocampal neurons in infantile rat hydrocephalus Turk Neurosurg 2011 21 3 352 358 21845571 10.5137/1019-5149.JTN.4119-11.1

[B48] Botsoglou N Fletouris DJ Papageorgiou GE Vassilopoulos VN Mantis AJ Trakatellis AG Rapid, sensitive, and specific thiobarbituric acid method for measuring lipid peroxidation in animal tissue, food, and feedstuff samples J Agric Food Chem 1994 42 1931 1937 10.1021/jf00045a019

[B49] Fisher M Feuerstein G Howells DW Hurn PD Kent TA Savitz SI Lo EH STAIR Group Update of the stroke therapy academic industry roundtable preclinical recommendations Stroke 2009 40 6 2244 2250 10.1161/STROKEAHA.108.541128 19246690 PMC2888275

[B50] (STAIR) STAIR Group Recommendations for standards regarding preclinical neuroprotective and restorative drug development Stroke 1999 30 2752 2758 10583007 10.1161/01.str.30.12.2752

[B51] Del Bigio M Slobodian I Schellenberg AE Buist RJ Kemp-Buors TL Magnetic resonance imaging indicators of blood–brain barrier and brain water changes in young rats with kaolin-induced hydrocephalus Fluids Barriers CNS 2011 8 22 10.1186/2045-8118-8-22 21834998 PMC3162928

[B52] Kannan R Kuhlenkamp JF Jeandidier E Trinh H Ookhtens M Kaplowitz N Evidence for carrier-mediated transport of glutathione across the blood–brain barrier in the rat J Clin Invest 1990 85 2009 2013 10.1172/JCI114666 1971830 PMC296671

[B53] Zhang Y Turunen M Appelkvist EL Restricted uptake of coenzyme Q is in contrast to unrestricted uptake of α-tocopherol into rat organs and cells J Nutr 1996 126 2089 2097 8814196 10.1093/jn/126.9.2089

[B54] Matthews R Yang L Browne S Balk M Beal F Coenzyme Q10 administration increases brain mitochondrial concentrations and exerts neuroprotective effects Proc Natl Acad Sci U S A 1998 95 8892 8897 10.1073/pnas.95.15.8892 9671775 PMC21173

[B55] Zhang Y Aberg F Appelkvist EL Dallner G Ernster L Uptake of dietary coenzyme Q supplement is limited in rats J Nutr 1995 125 446 453 7876919 10.1093/jn/125.3.446

[B56] Li H Klein G Sun P Buchan AM CoQ10 fails to protect brain against focal and global ischemia in rats Brain Res 2000 877 7 11 10.1016/S0006-8993(00)02609-3 10980237

[B57] Sikorska M Borowy-Borowski H Zurakowski B Walker PR Derivatised α-tocopherol as a CoQ10 carrier in a novel water-soluble formulation BioFactors 2003 18 173 183 10.1002/biof.5520180220 14695933

[B58] Sánchez-Moreno C Dorfman SE Lichtenstein AH Martín A Dietary fat type affects vitamins C and E and biomarkers of oxidative status in peripheral and brain tissues of golden Syrian hamsters J Nutr 2004 134 3 655 14988463 10.1093/jn/134.3.655

[B59] Kuwahara H Kanazawa A Wakamatsu D Morimura S Kida K Akaike T Maeda H Antioxidative and antimutagenic activities of 4-vinyl-2,6-dimethoxyphenol (canolol) isolated from canola oil J Agric Food Chem 2004 52 4380 4387 10.1021/jf040045+ 15237940

[B60] Torreilles F Salman-Tabcheh S Guerin M Torreilles J Neurodegenerative disorders: The role of peroxynitrite Brain Res Brain Res Rev 1999 30 153 163 10.1016/S0165-0173(99)00014-4 10525172

[B61] Szabo C The pathophysiological role of peroxynitrite in shock, inflammation, and ischemia-reperfusion injury Shock 1996 6 79 88 8856840 10.1097/00024382-199608000-00001

[B62] Quijano C Romero N Radi R Tyrosine nitration by superoxide and nitric oxide fluxes in biological systems: modeling the impact of superoxide dismutase and nitric oxide diffusion Free Rad Biol Med 2005 39 728 741 10.1016/j.freeradbiomed.2005.04.014 16109303

